# Polyamines (PAs) but not small peptides with closely spaced positively charged groups interact with DNA and RNA, but they do not represent a relevant buffer system at physiological pH values

**DOI:** 10.1371/journal.pone.0304658

**Published:** 2024-07-25

**Authors:** Julian Rieck, Christian Derst, Rüdiger W. Veh

**Affiliations:** Institut für Zell- und Neurobiologie, Charité - Universitätsmedizin Berlin, Berlin, Germany; Central University of Haryana School of Life Sciences, INDIA

## Abstract

Polyamines (PAs) including putrescine (PUT), spermidine (SPD) and spermine (SPM) are small, versatile molecules with two or more positively charged amino groups. Despite their importance for almost all forms of life, their specific roles in molecular and cellular biology remain partly unknown. The molecular structures of PAs suggest two presumable biological functions: (i) as potential buffer systems and (ii) as interactants with poly-negatively charged molecules like nucleic acids. The present report focuses on the question, whether the molecular structures of PAs are essential for such functions, or whether other simple molecules like small peptides with closely spaced positively charged side chains might be suitable as well. Consequently, we created titration curves for PUT, SPD, and SPM, as well as for oligolysines like tri-, tetra-, and penta-lysine. None of the molecules provided substantial buffering capacity at physiological intracellular pH values. Apparently, the most important mechanism for intracellular pH homeostasis in neurons is not a buffer system but is provided by the actions of the sodium-hydrogen and the bicarbonate-chloride antiporters. In a similar approach we investigated the interaction with DNA by following the extinction at 260 nm when titrating DNA with the above molecules. Again, PUT and tri-lysine were not able to interact with herring sperm DNA, while SPD and SPM were. Obviously, the presence of several positively charged groups on its own is not sufficient for the interaction with nucleic acids. Instead, the precise spacing of these groups is necessary for biological activity.

## Introduction

Polyamines (PAs) including putrescine (PUT), spermidine (SPD) and spermine (SPM) are small, versatile molecules with two or more amino groups and fulfil widespread biological functions [1]. Most living organisms require PAs for survival, but their precise biological functions are only partially understood.

Biological importance of PAs apparently is based on their molecular structures, consisting of two to four potentially charged amino groups with interspaced hydrophobic hydrocarbon chains. This composition offers, among others, two very likely functions: (i) Polyamines may act as potential buffer systems, and even in the absence of definitive evidence, many people are strongly convinced of this idea. (ii) Polyamines do interact with poly-negatively charged macromolecules, like (but not only) DNA and RNA.

A potential buffer function of PAs may be deduced from the fact that in some tumor associated cells they are used to maintain normal cytosolic pH [2]. In hemopoietic stem cells [3] as well as human leukemia cells [4] intracellular pH decreases, when PAs are depleted by administration of DFMO (alpha-difluoromethylornithine). Extracellular SPM is known to reduce the proton sensitivity of receptors inhibited by low pH [5].

Gene expression may be regulated by intracellular PAs in various ways [6]. PAs interact with DNA, influencing conformation and providing protection from endonucleases [7]. They bind to B-DNA preferably at the minor groove, seemingly depending on the nucleotide sequence [8].

Apparently, the presence of such simple molecules with multiple positively charged groups is required for the biological functions. The question arises, whether such compounds need to be PAs. Other simple molecules like small peptides with closely spaced positively charged side chains might be suitable as well. The same problem also holds for the interaction with nucleic acids. Are the detailed structures of SPD or SPM really essential for a functional interaction with DNA or RNA molecules?

The present report uses straightforward experiments to provide additional data for answering those questions. Consequently, the behavior of oligolysines (tri-Lys, tetra-Lys, and penta-Lys) was compared to that of PAs (PUT, SPD, and SPM) with respect to buffering capacity and specific interactions with DNA.

## Materials and methods

### Polyamine titrations

Polyamines (PAs) were obtained from Sigma-Aldrich Germany. Putrescine dihydrochloride (PUT), spermidine trihydrochloride (SPD) and spermine tetrahydrochloride (SPM) were each dissolved in 2 mM HCl, yielding a 10 mM solution of the respective PA at a pH-value below 3.0. 500 μl of each PA-solution was then titrated with 2.0 μl of a 500 mM NaOH solution until the pH exceeded a value of 10.0.

### Oligolysine titrations

Oligolysines (LysX) were obtained from Sigma Aldrich Germany. Tri-lysine (Lys3), tetra-lysine (Lys4) and penta-lysine (Lys5) were each dissolved in 5 mM HCl, resulting in a 10 mM solution at a pH-value close to 5.0. 500 μl of each LysX-solution was titrated with 2.0 μl of 500 mM NaOH until the pH-value exceeded 11.0.

### Spectroscopic analysis of polyamine DNA interaction

500 ng/μl herring sperm DNA (Sigma-Aldrich) in a physiological “intracellular” buffer (150 mM KCl, 10 mM NaCl, 10 mM HEPES, 2 mM MgCl_2_, pH 7.3) was mixed with various concentrations of spermine, spermidine, putrescine and tri-Lys (0–45 mM) in a total volume of 20 μl. Samples were incubated at 37°C for 15 min. Finally, absorption spectra between 220 nm and 320 nm were recorded on a TECAN Spark instrument (TECAN group, Männedorf, Switzerland) using the quartz optic NanoQuant plate. Spectra were analyzed using the Nano Quant App. Changes in the extinction at 260 nm (E_260_) were used to detect binding of PAs to DNA.

## Results

In general, the intracellular pH value is kept within narrow boundaries in eukaryotic cells [9, 10]. PAs might be an essential component of this process. The question arises, however, whether other simple molecules like small peptides with closely spaced positively charged side chains might be suitable as well.

### Polyamines or oligolysines as potential intracellular buffer systems

**Fig 1:**
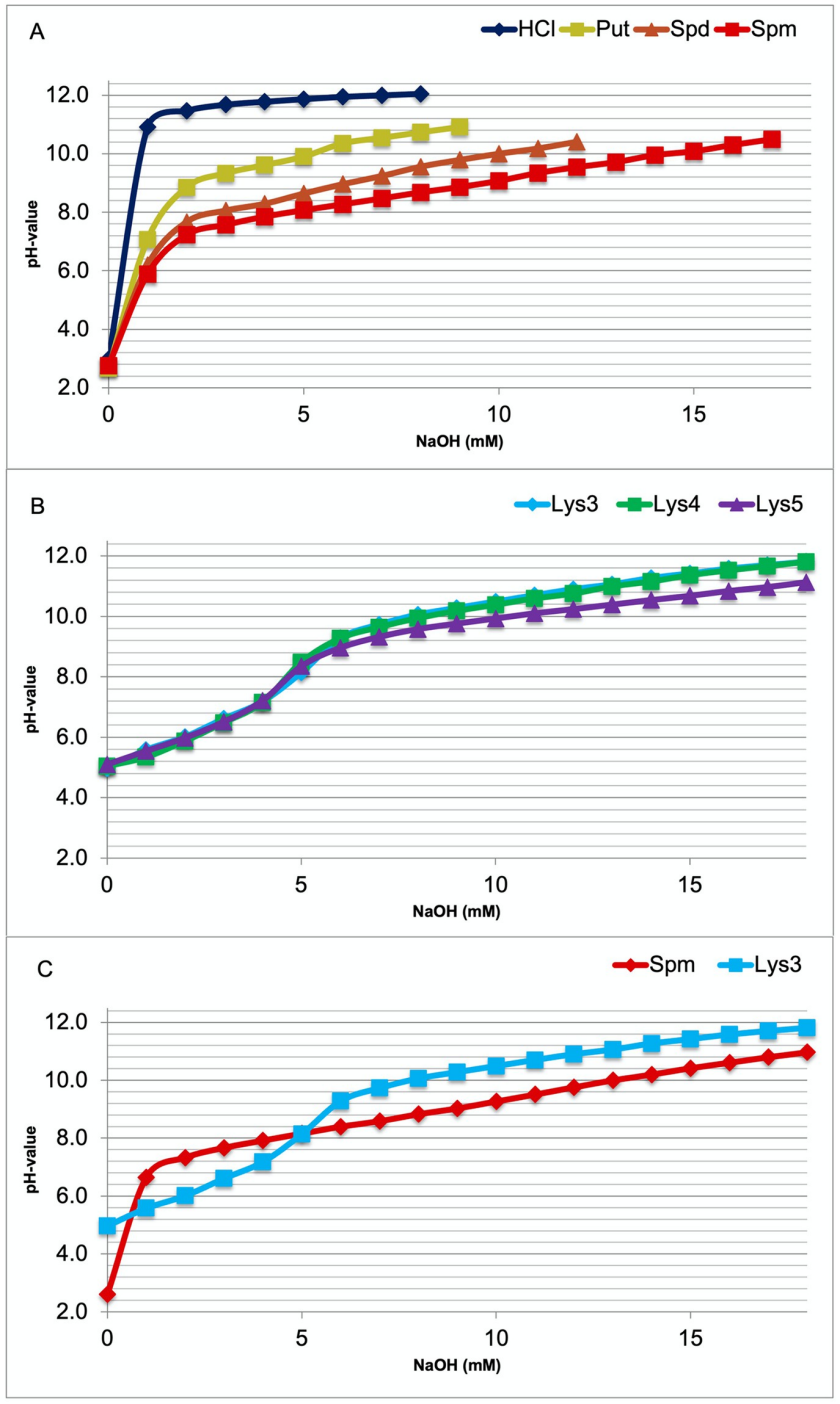
Comparative titration curves of HCl (control), polyamines, and oligolysines. Before titration all solutions were acidified to a value beneath the lowest pKa of the reactants. When comparing (1A) the polyamines putrescine (Put), spermidine (Spd), and spermine (Spm), the only compound that started buffering below a value of 7.4 was Spm. So, this is the only polyamine (PA), which to some degree may serve to stabilize the intracellular pH value within the physiological range. All the oligolysines (tri-lys, Lys3; tetra-lys, Lys4; penta-lys, Lys5) buffer the first time around pH 6.0 (alpha amino group) and secondly again above 8.0 far outside the physiological range (1B). In addition, the two molecules with four positive charges (Spm and Lys3) are compared directly (1C).

To obtain direct information on the pH range, where intracellular buffering of polyamines or oligolysines might occur, all the compounds were titrated to a pH value above ten. Titration curves of PAs (Fig 1A) display that buffering of PUT starts at about pH 8.4. Buffering by SPD already begins at about pH 7.6 and by SPM at about pH 7.2, close to their lowest pKa values (Table 1) as expected. Titration curves of tri-lysine (Lys3), tetra-lysine (Lys4), and penta-lysine (Lys5) all show a first buffering range around pH 6.0 (Fig 1B). Graphs are closer to each other, those of Lys3 and Lys4 rather identical. In the alkaline pH range buffering starts about 8.8 with Lys5 and about 9.2 with Lys3 and Lys4. The direct comparison between Lys3 and SPM titrations (Fig 1C) discloses that SPM is a much better buffer at close to neutral pH values.

**Table 1:** The pKs values of putrescine, spermidine, spermine, di-lysine, and tri-lysine as given here are taken from the investigations referenced. They are in good agreement with our data provided in the present manuscript.

Compound	pK1	pK2	pK3	pK4	pK5	reference
						
Putrescine	9,04	10,50	---	---	---	[11] Long et al,1961
						
Spermidine	8,25	9,71	10,90	---	---	[12] Kimberly & Goldstein,1981
						
Spermine	7,96	8,85	10,02	10,80	---	[13] Palmer et al, 1974
						
Lys-Lys	3,01	7,53	10,05	11,01	---	[14] Ellenbogen E, 1952
						
Lys-Lys-Lys	3,08	7,34	9,80	10,54	11,32	[14] Ellenbogen E, 1952

In most cells the intracellular pH is maintained in the range from 7.0 to 7.4 [9]. A very similar value (7.03 to 7.46) is also found in hippocampal neurons [10]. As seen from above (Fig 1), SPM is the only compound, which may directly contribute to stabilizing the intracellular pH in the required range. Surprisingly, the oligolysines are not suitable for this purpose. Their carboxyl groups buffer around 3.0, their alpha amino groups around 6.0, and their epsilon amino groups above 8.5, all outside the physiological boundaries (see Table 1).

### Polyamines or oligolysines as potential interactants with nucleic acids

PAs are well known to interact with DNA and RNA [15–17] among other polyanionic molecules. The interaction with nucleic acids is of utmost biological importance. It influences the conformation of DNA molecules, determines the density of packed DNA or regulates the translation of RNA molecules [6–8, 16, 17]. Again, the question arises, whether the detailed structures of SPD or SPM are vital for this functional interaction, or whether oligolysines could serve as potential alternatives.

**Fig 2:**
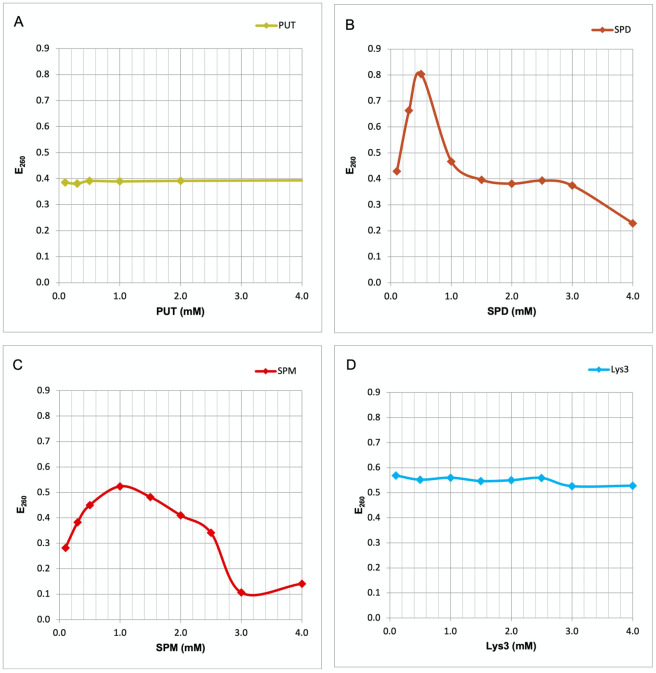
Comparative interaction curves polyamines and tri-lysine with DNA. In the present study we measured changes in the UV absorption at 260 nm (E_260_) of herring sperm DNA in the presence of PAs or tri-lysine (Lys3). Addition of PUT, the smallest PA with only two charged groups, did not produce any alteration in the E_260_ of the DNA (Fig 2A). In contrast, SPD and SPM appear to interact with the DNA, as both PAs strongly heighten E_260_ values. With SPD the increase is highest at 0.5 mM, followed by a steep decrease in extinction. This decrease, however, must not be interpreted as conformational change but is simply the result of DNA precipitation at higher SPD concentrations (Fig 2B). With SPM the maximum increase occurs at 1 mM (Fig 2C), followed by precipitation. Again, the elevated E_260_ values at SPM concentrations above 1 mM may be due to turbidity produced by the precipitate. Addition of Lys3, which like SPD bears three positively charged groups, results in no increase at E_260_ (Fig 2D), even at concentrations up to 45 mM. Thus, only the longer PAs like SPD and SPM, and neither PUT nor Lys3 are able to interact and produce conformational changes in double stranded DNA molecules.

Conformational changes of nucleic acids may be recognized by UV spectroscopy [18]. Consequently, in the present study we measured changes in the UV absorption at 260 nm (E_260_) of herring sperm DNA in the presence of PAs or tri-lysine (Lys3). Addition of PUT, the smallest PA with only two charged groups, did not produce any alteration in the E_260_ of the DNA (Fig 2A). In contrast, SPD and SPM appear to interact with the DNA, as both PAs strongly heighten E_260_ values. With SPD the increase is highest at 0.5 mM, followed by a steep decrease in extinction. This decrease, however, must not be interpreted as conformational change. It simply is the result of DNA precipitation at higher SPD concentrations (Fig 2B), which also is observed visually. With SPM the maximum increase occurs at 1 mM (Fig 2C), again followed by precipitation. The elevated E_260_ values at SPM concentrations above 1 mM may be due to turbidity produced by the precipitate. Interestingly, addition of Lys3, which like SPD also bears three positively charged groups, results in no increase at E_260_ (Fig 2D), even at concentrations up to 45 mM. This fact indicates that Lys3 is not able to interact with the DNA molecule. Taking together, only the longer PAs like SPD and SPM, and neither PUT nor Lys3 are able to interact and produce conformational changes in double stranded DNA molecules.

## Discussion

Biologial functions of PAs are largely based on their molecular structure with two to four charged amino groups and interspaced hydrophobic hydrocarbon chains. These structures suggest are two likely biological functions: (i) as potential buffer systems and (ii) as interactants with poly-negatively charged nucleic acids, proteins, or lipid complexes.

### Contribution of PAs to extracellular pH homeostasis is barely relevant

PH homeostasis in the mammalian body requires at least two separate systems: one maintaining the intracellular H^+^ concentration and another one keeping the extracellular value in the physiological range. Both systems are interconnected by the sodium-hydrogen antiporter [19] and the bicarbonate-chloride antiporter [20].

Homeostasis of the extracellular pH value in the body is largely provided by the kidney and the lung. In the brain, however, there is an additional player, the astrocyte [21]. Especially in the brainstem astrocytes are able to sense decreases in the pH as small as 0.2 units in the extracellular pH-value [22]. Subsequent to extracellular acidification by increased neuronal activation about 30% of all astrocytes release bicarbonate to buffer the interstitial space [23]. This release is mediated by the sodium bicarbonate transporter NBCe1 (Natrium Bicarbonate Cotransporter electrogenic 1; SLC4A4), which is highly expressed in astrocytes [23]. Subsequent to conditional knockdown of this NBCe1 transporter in mice the extracellular pH in the brain unstable and increased neuronal activity results in significant extracellular acidification [23].

### PAs do contribute to intracellular pH homeostasis

PAs often are considered as potential buffer systems to directly stabilize the intracellular pH value [2, 4]. This, however, appears rather unlikely from our data (Fig 1), as the only PA is SPM, which only is able to exert minor buffering capacity at intracellular pH values.

### Indirect contribution of PAs to intracellular pH homeostasis

**Fig 3:**
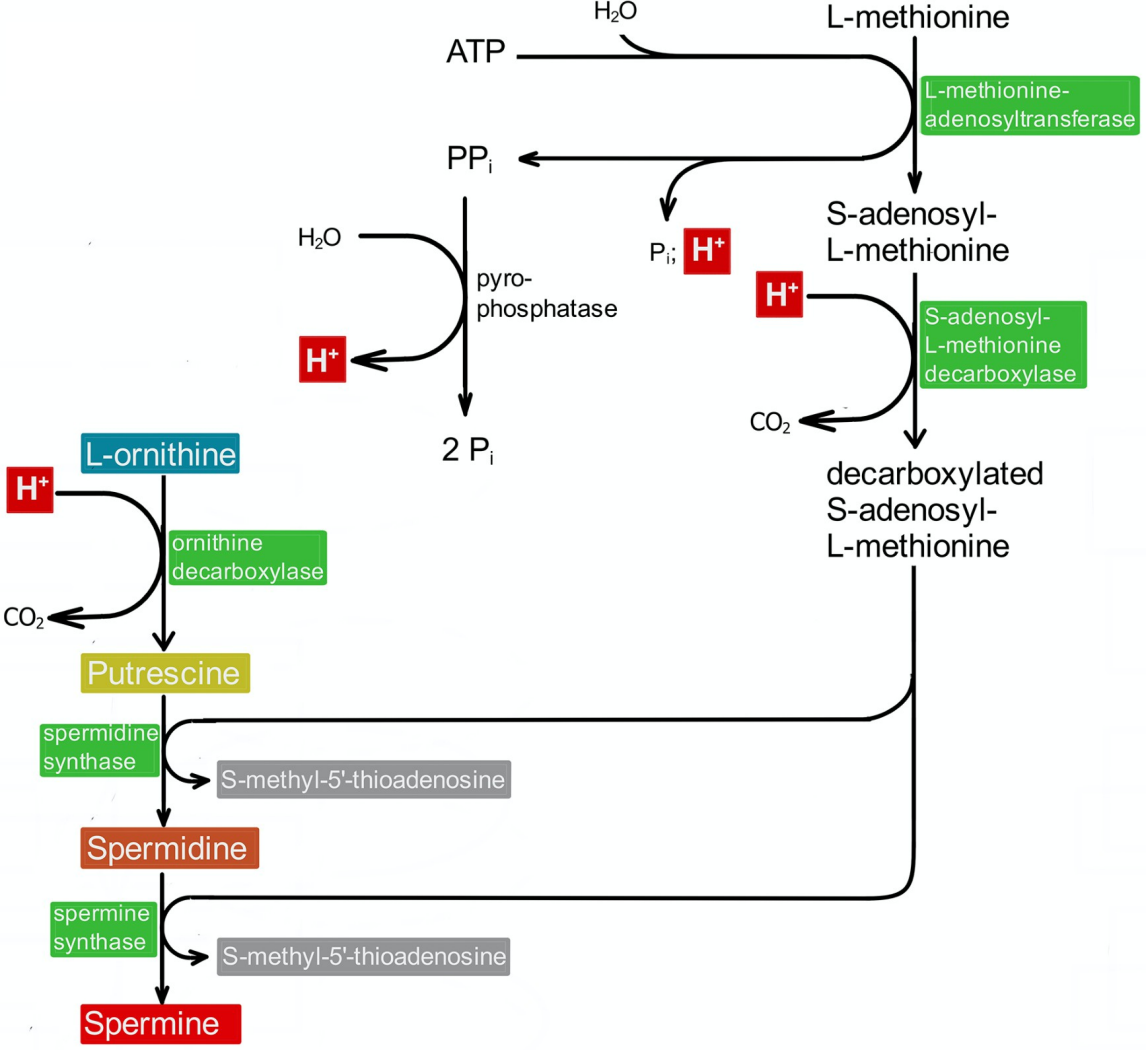
Indirect contribution of PAs to intracellular pH homeostasis. PAs may indirectly contribute to the stabilization of the intracellular pH value, as their biosynthesis includes several steps, which are thought to increase the intracellular pH value. The present scheme is an extract of our earlier one (20: Rieck et al, 2022, Fig 2) representing the biosynthesis of PAs from ornithine. Here, we focus on reactions, which release H^+^-ions (highlighted by red boxes) into the cytoplasm. Viewing this scheme, however, one should bear in mind that the biosynthesis of S-adenosylmethionine (methionine plus ATP releases three inorganic phosphates) should produce more H^+^-ions than had been consumed before.

PAs, however, may indirectly contribute to the stabilization of the intracellular pH value. The biosynthesis of PAs includes several steps, which are thought to increase the intracellular pH value. Enzymes like S-adenosylmethionine decarboxylase and ornithine decarboxylase (ODC) consume H^+^-ions (see Fig 3). Consequently, blocking their activity as shown in the case of ODC should decrease the intracellular pH value, and this is actually the case [2, 4]. Restoring PA synthesis in these cells by the addition of PUT leads to an increase of the pH value again [2]. This effect may be due to the consumption of two more protons, as the decarboxylation of S-adenosymethionine (SAM) requires one proton, and two molecules of decarboxylated SAM are necessary for the biosynthesis of SPM (Fig 3). These reflections, however, may be questionable. When the production and the necessary amount of SAM is included in the consideration it turns out that its biosynthesis (methionine plus ATP yields SAM plus three inorganic phosphates) should produce more H^+^-ions than had been consumed before.

### Proteins contribute to intracellular pH homeostasis

In addition to small molecular buffers like bicarbonate and phosphate, the cell contains a very large amount (20–30%) of proteins [24]. The corresponding concentrations are estimated to 160 to 310 mg/ml [25]. Assuming an average molecular weight of 50 kD and a 10% share of side chain carboxylated amino acids (aspartate or glutamate) for cellular proteins [26], this results in a buffering capacity of about 20 to 30 mM, which is not that much but more that the potential contribution of SPM (intracellular 1 mM [27]) and may be fundamental.

### Proton transport is the most important mechanism for intracellular pH homeostasis

The most important mechanism of intracellular pH homeostasis in neurons [10] is represented by the metabolic generation of H^+^ ions [28, 29], the effect of the sodium hydrogen antiporter (NHE1 or SLC9A1 [19, 30]), and the action of the bicarbonate-chloride antiporter (AE3 or SLC4A3 [20, 31]). The NHE1 protein possesses a characteristic intracellular domain, which is phosphorylated, when the internal pH-value is decreased below a given set point. Then the transporter activity is increased, and thereby the set point defines the actual internal pH value of the neuron [32].

In astrocytes intracellular pH is predominantly maintained by the NBCe1 transporter (Natrium Bicarbonate Cotransporter electrogenic 1; SLCA4A), which also influences the extracellular pH in the brain [21]. PAs are believed to exert direct action on the NHE1 set point thereby regulating the intracellular pH value [4]. Most likely, therefore, the role of PAs in intracellular pH homeostasis is not due to a direct or indirect buffering mechanism, but on their interaction with the sodium-hydrogen antiporter system.

## Conclusion

In this work we investigated two plausible functions of Polyamines (PAs): (i) as potential buffer systems and (ii) as interactants with poly-negatively charged nucleic acids. The report focuses on the question, whether PAs are essential for the assessed functions, or whether other simple molecules like small peptides with closely spaced positively charged side chains might be suitable as well.

In fact, no members of these two groups are optimal for intracellular buffering at physiological pH values. The in vivo contribution of PAs to the stability of the intracellular pH values, therefore, is more likely due to their effect on the set point of the sodium hydrogen antiporter system.

In contrast, SPD and SPM, but not PUT or oligolysines are able to interact with DNA molecules. Obviously, the precise spacing of the positively charged groups is important for biological activity. The present observations contribute to the growing insight into specific roles of PAs in cellular homeostasis and emphasize the importance of their distinct molecular structures.

## Supporting information

S1 File (XLSX)

S2 File (XLSX)
